# Acute Doxorubicin Insult in the Mouse Ovary Is Cell- and Follicle-Type Dependent

**DOI:** 10.1371/journal.pone.0042293

**Published:** 2012-08-02

**Authors:** Elon C. Roti Roti, Scott K. Leisman, David H. Abbott, Sana M. Salih

**Affiliations:** University of Wisconsin-Madison, Department of Obstetrics and Gynecology, Reproductive Endocrinology and Infertility Division, Madison, Wisconsin, United States of America; Imperial College London, United Kingdom

## Abstract

Primary ovarian insufficiency (POI) is one of the many unintended consequences of chemotherapy faced by the growing number of female cancer survivors. While ovarian repercussions of chemotherapy have long been recognized, the acute insult phase and primary sites of damage are not well-studied, hampering efforts to design effective intervention therapies to protect the ovary. Utilizing doxorubicin (DXR) as a model chemotherapy agent, we defined the acute timeline for drug accumulation, induced DNA damage, and subsequent cellular and follicular demise in the mouse ovary. DXR accumulated first in the core ovarian stroma cells, then redistributed outwards into the cortex and follicles in a time-dependent manner, without further increase in total ovarian drug levels after four hours post-injection. Consistent with early drug accumulation and intimate interactions with the blood supply, stroma cell-enriched populations exhibited an earlier DNA damage response (measurable at 2 hours) than granulosa cells (measurable at 4 hours), as quantified by the comet assay. Granulosa cell-enriched populations were more sensitive however, responding with greater levels of DNA damage. The oocyte DNA damage response was delayed, and not measurable above background until 10–12 hours post-DXR injection. By 8 hours post-DXR injection and prior to the oocyte DNA damage response, the number of primary, secondary, and antral follicles exhibiting TUNEL (terminal deoxynucleotidyl transferase dUTP nick end labeling)-positive granulosa cells plateaued, indicating late-stage apoptosis and suggesting damage to the oocytes is subsequent to somatic cell failure. Primordial follicles accumulate significant DXR by 4 hours post-injection, but do not exhibit TUNEL-positive granulosa cells until 48 hours post-injection, indicating delayed demise. Taken together, the data suggest effective intervention therapies designed to protect the ovary from chemotherapy accumulation and induced insult in the ovary must act almost immediately to prevent acute insult as significant damage was seen in stroma cells within the first two hours.

## Introduction

With improvements in cancer therapy, women with cancer survive their initial diagnosis in ever greater numbers. This continual increase in cancer survivorship makes it necessary to develop simple and effective approaches to limit unintended consequences of chemotherapy, including primary ovarian insufficiency (POI) and infertility. POI occurs in up to 40% of reproductive age breast cancer survivors and over 8% of childhood cancer survivors, who will make up 1 in 800 women by the year 2020 [1], [2], [3], making chemotherapy-induced POI an imminent challenge facing the medical community. Premature menopause in turn increases the patient’s risk for subsequent complications, including osteoporosis, infertility, and cardiovascular disease. POI has long been documented as a consequence of chemotherapy, but the acute stages of toxicity are not well-understood, hampering efforts to prevent ovarian demise. Protecting the ovary from unintended chemotherapy damage first requires an overall understanding of the ovarian cell types targeted by chemotherapy, the chemotherapy’s mode of action, and the acute timeline of insult.

Defining chemotherapy insult is complex given the heterogeneous nature of the ovary which is composed primarily of stroma cells and follicles. The follicles are in turn specialized layers of theca cells (derived from stroma) and granulosa cells that surround and nourish the oocyte. The stroma and theca cells are the only ovarian cells in direct contact with systemic circulation. Numerous studies have shown follicle and oocyte attrition following chemotherapy [4], [5], [6], [7], [8], [9], [10], [11], but it is unclear whether oocytes are directly targeted by the anti-cancer process, or deteriorate as the surrounding follicular cells fail.

Doxorubicin (DXR), an anthracycline, was first used in clinical trials in the 1960’s and is still a cornerstone agent in frontline chemotherapy regiments, currently used to treat roughly 50% of all cancer cases occurring in premenopausal females, including breast and childhood cancers [12], [13], [14], [15]. DXR can cause double-strand DNA breaks in a topoisomerase II-dependent manner or induce oxidative stress depending on the cell type and drug dose. The cellular response to the DXR insult is also cell type- and dose-dependent. A single cell line can respond to different doses of DXR by committing to apoptosis, induced cell cycle arrest, senescence, autophagy, or necrosis [16]. Although the long-term morphological effects of DXR on the ovary have been documented, the mode(s) of oocyte and follicle demise are not well understood [16], [17], [18], [19], [20], [21], [22], [23]. Previous studies have demonstrated DXR treatment causes apoptosis and follicular attrition as early as 12 hours post-injection in mice [22], [23], [24], followed by partial recovery (1 month post-DXR), when regular ovulation recovers to 50% of the pre-DXR ovulation rate despite permanent reduction of ovary size by 50% [25]. Oocytes directly exposed to DXR *in vitro* can undergo DXR-induced oxidative stress [26] and exhibit chromosome condensation and altered gene expression [27], [28]. Which ovarian cell and follicle types are primary targets of DXR *in vivo*, however, is not understood.

DXR is autofluorescent, allowing direct monitoring of the drug’s penetration in the ovary. DXR has a unique fluorescent fingerprint; upon excitation at 488 nm, the drug produces fluorescence emissions from 525–700 nm [29], [30], [31], [32], [33], [34], [35], [36], [37], [38], [39], [40], [41], [42]. This spectral profile (fingerprint) can shift in response to environment, including changes in pH and aqueous vs. lipid environments [29], . Peak shifts in DXR’s spectral profile to shorter wavelengths, or blue shifts, are associated with a solvent environment with a low dielectric constant and/or higher pH, while shifts to red (longer) wavelengths are associated with an environment with a higher dielectric constant and/or lower pH [30], [43]. DXR’s spectral emission profile therefore acts as a reporter of subcellular environment.

The goal of this study is to define acute DXR accumulation and DNA insult in the mouse ovary, identify target cells, and a define window of opportunity prior to the apoptotic response to target future acute chemoprotection strategies. Using DXR’s autofluorescence, this study defines the accumulation of DXR in the mouse ovary as beginning in the central stroma tissue and radiating outward over time as DXR penetrates into the follicles and the cortex. Quantifying double strand DNA breaks using the comet assay revealed that acute DNA damage following DXR injection occurred first in stoma cells (2 hours post-injection), followed by a robust DNA damage response in granulosa cells (4 hours post-injection). DXR-induced follicular apoptosis plateaued subsequent to granulosa DNA damage, at 8 hours post-DXR injection, prior to the time points at which DNA damage rises above background in oocytes. Primordial follicles exhibited apoptotic signals and phospho-H2AFX staining indicating DNA damage only at 48 hours post-DXR injection, a delayed demise compared to growing follicles. The current study provides a robust framework in which future studies can test the efficacy of potential ovarian protective agents and suggests chemoprotectants should be immediately active (within two hours post-injection) to prevent DXR toxicity. In addition, this study highlights the heterogeneous response of the ovary to DXR and suggests depleting the ovarian reserve may be due to the combination of ovarian stroma demise, increase recruitment of primordial follicles following burn out of growing follicles, and a delayed damage response in the primordial follicles.

## Materials and Methods

### Mice

#### Statement of ethical approval

This study was conducted in strict accordance with the Guide for the Care and Use of Laboratory Animals and the Animal Welfare Act and its subsequent amendments and procedures were approved by the Medical School Animal Care and Use Committee of the University of Wisconsin-Madison (permit number M02213-0-07). Animals were housed in the University of Wisconsin Animal Care Facility, accredited by the Association for Assessment and Accreditation of Laboratory Animal Care, and provided a standard care with free access to food and water. Female CD1 mice were purchased from Charles River Laboratories, Wilmington MA through the University of Wisconsin Animal Care Facility. Mice were utilized at 4-weeks old as a model for older children/adolescents with cancer, a patient population with a relatively high survival rate, which will face potential reproductive failure with grave consequences including failure of pubertal development following chemotherapy. Four-week old female CD1 mice were treated with 20 mg/kg doxorubicin (twice the human equivalent dose to allow visualization and quantification of DXR autofluorescence) or saline via intraperitoneal injection (200 µL total volume/injection). Two to four mice were injected per time point, 2, 4, 6, 8, 10 12, 24, and 48 hours, depending on the experiment. The time points were staggered such that mice from all time points and control were euthanized with CO_2_ side-by-side per approved protocol and every effort was made to minimize suffering. Ovaries were removed surgically, placed in 2 mL of DMEM/F-12 50/50 media (minus phenol red, Mediatech), and cleansed of bursa and attached fat. One ovary from each mouse was fixed with 10% formalin and processed for fluorescence microscopy (immunofluorescence or TUNEL assay, see below). The second ovary from each mouse was processed for the comet assay (below) to provide paired data.

### Neutral Comet Assay

Ovaries were processed to provide enriched populations of granulosa cells mixed with oocytes, and stroma/theca cells as previously described [47]. Briefly, the granulosa cells and oocytes released by puncturing follicles were resuspended in 100 µL 0.1% proteinase K in PBS (phosphate buffered saline: 137 mM NaCl, 2.7 mM KCl, 10 mM Na_2_PO_4_, 1.76 mM KH_2_PO_4_) and incubated 5 min at room temperature (RT) to digest the zona pelucida surrounding oocytes. Spent ovarian tissue containing stroma, theca, and any residual granulosa cells, was treated with 0.25% collagenase IV in PBS as previously described [47]. These samples, enriched for stroma/theca cells, were passed through a 23-gauge needle five times and samples were blinded. The two separate, enriched pools of cells (granulosa/oocytes and stroma/theca) were then blinded and processed for the neutral comet assay [47]. For simplicity, the enriched cellular fractions will be referred to as “granulosa,” “oocytes,” or “stroma/theca” cells throughout the study. The method used favors collection of granulosa cells from antral follicles, as those from primordial follicles are not expected to release following ovarian puncture. Granulosa cells from the primordial and primary follicles are typically not obtained by puncturing the antral follicles and are expected to be present in the “stroma/theca” enriched cellular fractions. Images were collected on an Olympus microscope using a 20X objective and SPOT Plus software. We conducted four replicates with two to four mice per replicate. At least 100 cells per time point per mouse were imaged for the granulosa cells and stroma/theca cells, and 50 oocytes per time point per mouse. The Olive Moment (OM) for comets was scored using CometScore software (TriTek Corporation). Statistical significance was determined using a one-way ANOVA with Bonferroni means comparison at p<0.05. Data were normalized to control for each experiment to allow pooling across experiments.

**Figure 1:**
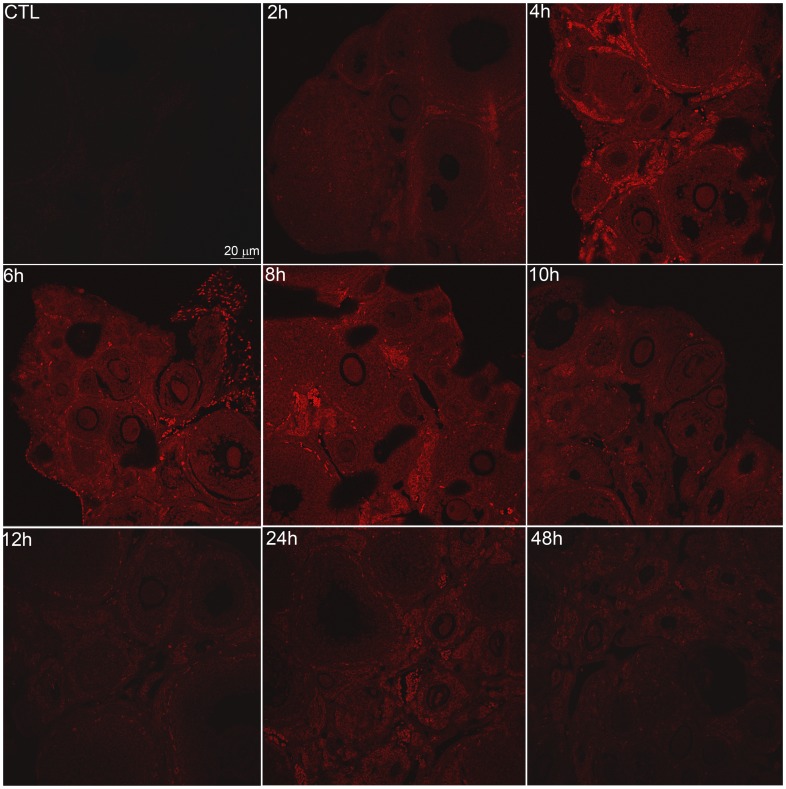
DXR fluorescence was visible in the ovary. Representative confocal images of DXR signal in ovarian sections reveal time-dependent penetration of DXR into the ovary. Images from control and mice treated for 2, 4, 6, 8, 10 12, 24 hours with 20 mg/kg were taken using a 20X objective, exciting at 488 nm and collection emissions from 570–620 nm (final magnification  = 200X). All images were adjusted +10 brightness and +10 contrast to enhance visibility in print. Scale bar  = 20 µm.

### Histology and Fluorescence Microscopy

Images were collected using a Nikon A1 laser scanning microscope (W.M. Keck Laboratory for Biological Imaging, UW-Madison). For imaging DXR via standard confocal microscopy based on its autofluorescence, a 488 nm laser was used for excitation, and emissions were collected in the red channel (band pass wavelengths 570–620). Laser settings were determined by imaging control samples and setting laser intensities such that autofluorescent signal was barely detectable. Settings were then held constant used for each DXR-treated sample. Each image was taken at the Z plane providing maximal signal in the given section. Spectral images were acquired in a similar fashion, but utilized the spectral scan head on the A1 confocal, exciting at 488 nm and collecting emissions from 520 nm through 720 nm at 10 nm intervals. For γH2AFX phosphorylation and TUNEL assay, ovarian sections were imaged with a Nikon A1 confocal microscope. For DAPI (4′,6-diamidino-2-phenylindole), FITC, and PI (propidium iodide)-stained images excitation/emission wavelengths were 403.6/450, 488/525, and 561/570-620, respectively. Merged and individual channels were collected.

**Figure 2:**
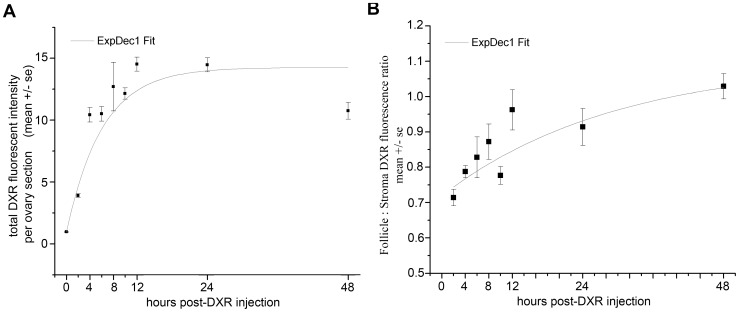
DXR accumulation in the ovary and follicle was time-dependent. *A.* Graph of total DXR fluorescence intensity in the ovary as a function of time reveals DXR accumulation in the ovary follows an exponential fit, with signal rapidly rising through 4 hours post-injection, followed by plateau (n = 4). *B.* The ratio of DXR in follicles vs. stroma was calculated as a function normalized to area. The graph demonstrates the ratio of DXR fluorescence in follicles vs. stroma increases steadily over time (n = 4).

### Fluorescence Quantification

#### DXR fluorescent intensity

Total DXR fluorescence was measured in each section image using Image J, subtracting any areas of the image that did not contain ovarian tissue and any blood vessels/red blood cells to ensure only DXR signal and areas corresponding to ovarian tissue was measured. DXR fluorescence was then measured either as total intensity or as mean intensity (total fluorescent intensity divided by the area).

**Figure 3:**
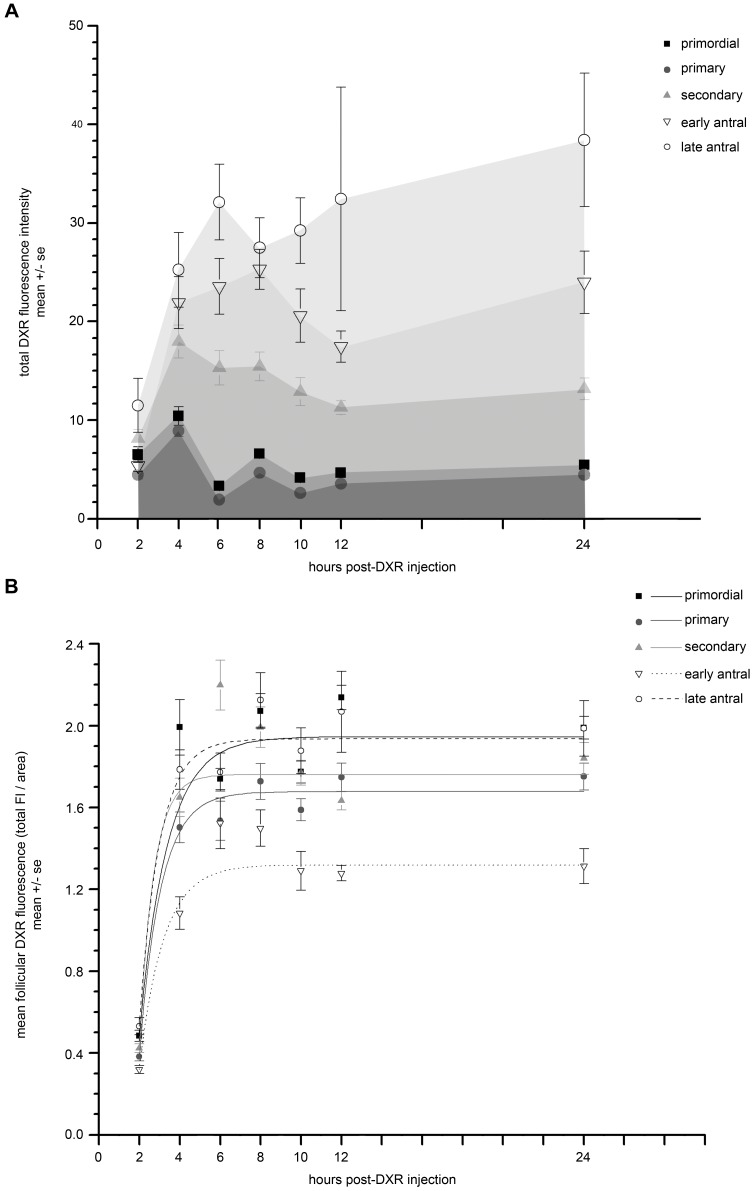
DXR accumulation differed according to follicle class. *A.* Graph depicts mean total DXR fluorescence intensity for each follicle class (primordial, primary, secondary, early antral, late antral) plotted over time. All follicle classes exhibited a rapid rise in mean fluorescent signal over the first 4 hours, but the larger follicles had higher total fluorescence levels than smaller follicles. *B.* Graph depicts mean follicular DXR fluorescence (total fluorescent intensity/area) plotted as a function of time. Normalizing to area in this manner minimizes differences in the number of granulosa cells/follicle to discern DXR accumulation differences that are a function of follicle class rather than cell number. Mean DXR accumulation in primordial and late antral follicles was indistinguishable, but significantly greater than primary, secondary, and early antral follicles (p≤0.05, two-way ANOVA; 6 sections per time point; n = 3 animals for quantification). Mean DXR accumulation in primary, secondary, and early antral follicles is also significantly distinct for each follicle class (p≤0.05, two-way ANOVA).

**Table 1:** exponential decay fits, y = A1*exp(-x/t1)+y0.

	primordial		primary		secondary		early antral		late antral		total ovary fl	
	value	se	value	se	value	se	value	se	value	se	value	se
**y0**	1.94487	0.0972	1.67659	0.04279	1.76051	0.08023	1.31869	0.04807	1.93459	0.07029	14.28223	1.6808
**A1**	−6.30796	5.69E+00	−8.63099	4.61908	−22.8023	76.15835	−5.39344	3.69796	−10.7129	7.42542	−13.2983	1.68142
**t1**	1.36641	0.87076	1.05457	0.30428	0.7053	0.83503	1.18696	0.49344	0.98421	0.34801	6.11285	1.5934

To measure the amount of DXR fluorescent signal according to follicular stage, each follicle was selected as a region of interest and the corresponding total fluorescent intensity units and area were measured for each follicle. To quantify mean total DXR fluorescence per follicle class, at least 50 follicles from each class were quantified from at least 6 sections (representing first third, middle, and last third of each ovary), with ovaries from 3 mice per n. Follicles were classified mainly according to morphology, in addition to size as follows [48]: primordial 12–23 µm, primary 20–49 µm, secondary 50–103 µm, early antral 104–234 µm with ≤50% antral space, late antral 84–452 µm. Each follicle per image section was measured and classified with a specific stage or N/C =  not classified. DXR follicular signal was subtracted from the total DXR signal per image to give the total DXR fluorescence present in stroma cells. DXR fluorescence was not measured on an individual follicle level at 48 hours post-injection because the deterioration of follicles made classification unreliable. Follicle/stroma fluorescence ratio was quantified as: (follicle intensity/follicle area)/(stroma intensity/stroma area).

**Figure 4:**
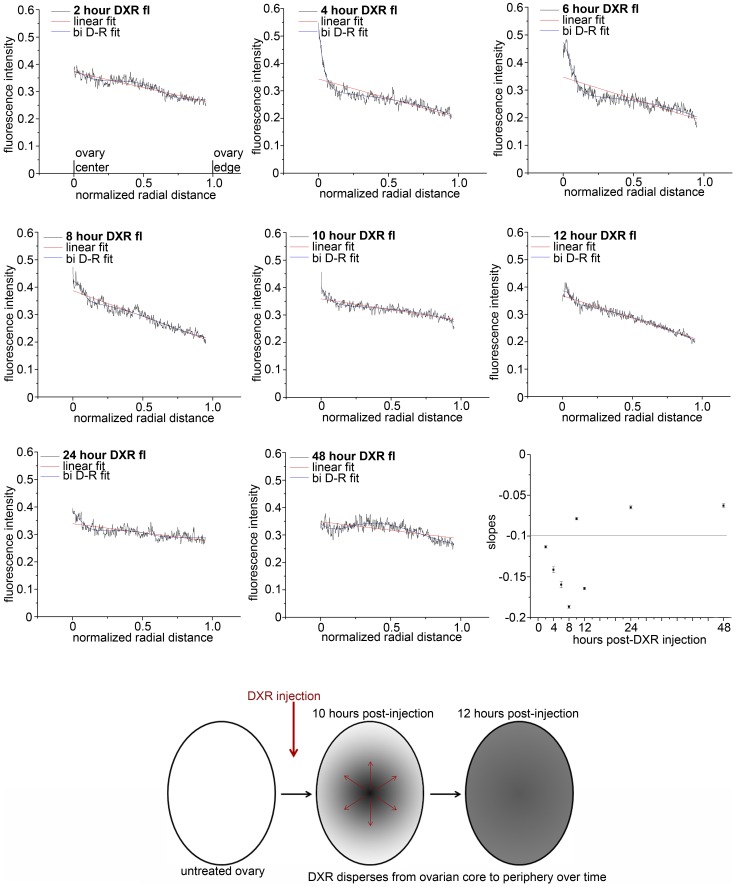
Radial distribution of DXR in the ovary changed over time. Graphs summarize radial distribution of DXR fluorescence from the center to the perimeter of ovarian sections at each time point (≥3 sections per time point; n = 3 animals for quantification). On the X axis, zero represents the center of the ovary, and 1 is the periphery of the ovary as indicated on the 2 h graph. Linear fit lines are red and bi-dose response curve fits are blue. Cartoon models DXR distribution change over time.

**Table 2:** Linear slope and R^2^ values for DXR distribution across the ovary change over time.

time (hours) post-DXR injection	linear slope	R∧2 linear fit	R∧2 biDoseResponse fit
**2 hours**	−0.11341	0.8449	0.89507
**4 hours**	−0.14133	0.63132	0.938
**6 hours**	−0.1597	0.64329	0.92476
**8 hours**	−0.18689	0.91411	0.94789
**10 hours**	−0.07875	0.75064	0.84038
**12 hours**	−0.16444	0.94886	0.95733
**24 hours**	−0.06489	0.56965	0.72432
**48 hours**	−0.06271	0.43415	0.73334

#### Radial distribution

To define the radial distribution of DXR in images of ovarian slices, lines were drawn from the center of the ovary to its extremity and the peak profile along each line was generated in Image J. Lines were drawn at every hand of the clock in each image, provided there were no folds in the tissue and the perimeter of the ovary could be seen. If only part of the ovarian slice fit within the field of view, lines were taken at each clock hand possible. The peak profiles for each line were normalized on a scale of 0 to 1 for distance and 0 to 1 for DXR fluorescent intensity. This allowed pooling of the lines to compare distribution as a function of ovarian center to extremity. Lines generated from 3 slices each from 3 different animals were averaged using OriginLab to generate the radial distribution curves. The distance values were truncated at 0.95 to eliminate operator error in defining the exact perimeter of the ovary.

**Figure 5:**
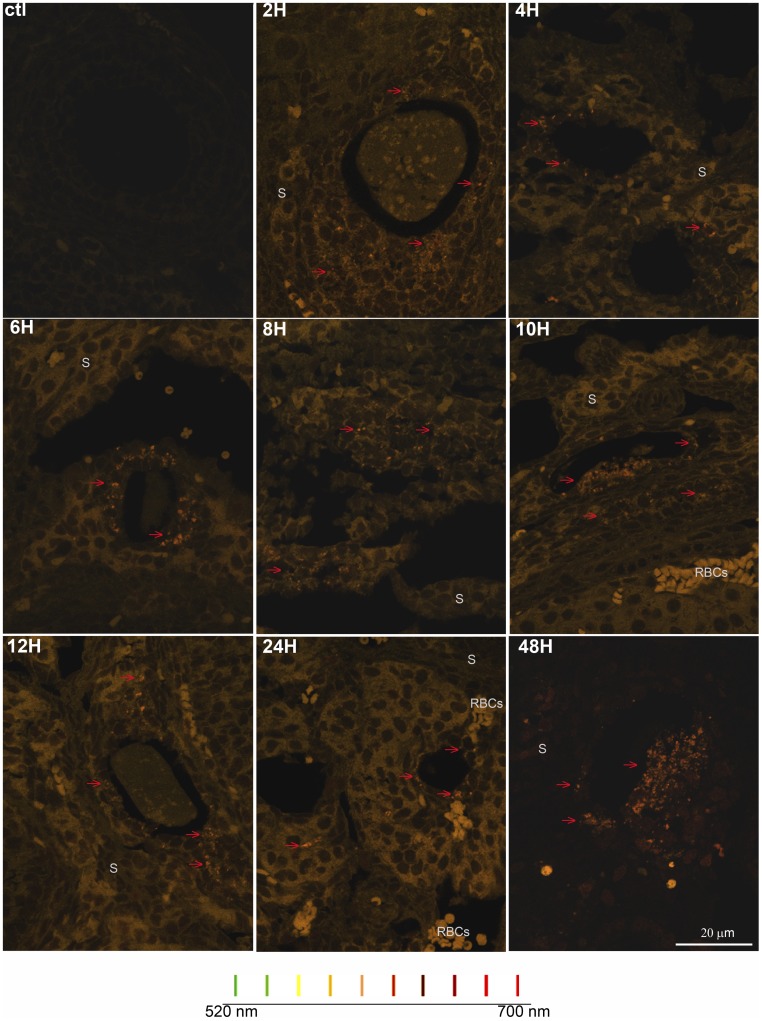
DXR spectral emissions revealed cell-type dependent subcellular accumulation. Example spectral composite confocal images are shown for each time point. Images were generated using laser scanning spectral confocal microscopy, exciting at 488 nm and collecting the emissions images every 10 nm from 520 to 700 nm using a 60X objective (600X final magnification). Colored bars show how each wavelengths is represented by a different color coding; all wavelengths are represented in composite images. All images were adjusted +10 brightness and +10 contrast to enhance visibility in print. Scale bar  = 20 µm.

### Phospho-H2AFX Staining

Ovary sections on slides were deparaffinized, hydrated, and rinsed for 5 min in distilled-deionized H_2_O. Sections were blocked for 1 hour at RT using 10% goat serum in PBS, followed by a PBS wash. Sections were then incubated with rabbit anti-phospho-H2AFX (Cell Signaling) at a 1∶480 dilution in PBS with 1% goat serum overnight at 4°C. Sections were rinsed 3 times for 5 minutes in PBS, then incubated in goat anti-rabbit Alexafluor 488 (Invitrogen) at a 1∶400 dilution in PBS for 30 minutes at RT in the dark. Slides were rinsed 3 times for 5 minutes in PBS. Coverslip was placed with Crystal Mount containing 1 µg/mL propidium iodide or ProLong Gold antifade reagent with DAPI (Invitrogen).

**Figure 6:**
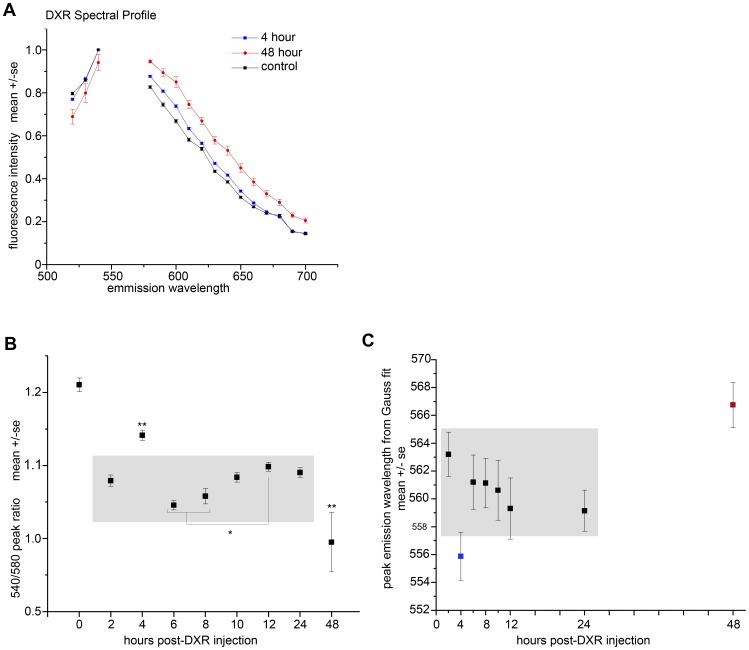
DXR’s spectral profile in the ovary shifted over time. *A.* Graph plots spectral fluorescence intensity values for control ovary images and images from mice treated with DXR for 4 and 48 hours. DXR signal in sections from 4 and 48 hours exhibited a spectral profile different from control; DXR spectral profiles for all time points were different from control (2,6,8,10,12, and 24 hours in Supplemental Figure S5). The gap in the connecting lines corresponds to the “cold finger” (550–570 nm), or the wavelengths over which the A1 confocal collects no spectral data. *B.* Graph plots the fluorescent intensity ratio for 540/580 nm versus time, revealing changes in the peak intensity ratio over time post-DXR injection (one-way ANOVA, p≤0.05, **sig. vs. all points, *sig. vs. 12 hour point). *C.* Graph plots wavelength peaks generated by a Gauss fit of the spectral curve at each time point. Plot reveals shifts in the apparent peak over time post-DXR injection (see Supplemental Figure S5 for graphs with fit curves).

### TUNEL Staining

Apoptosis was detected in ovarian sections utilizing ApopTag Plus Fluorescein In Situ Apoptosis Detection Kit; all solutions mentioned were supplied with the kit (Chemicon International Cat. No. S7111). Immunoflourescent staining was performed per kit instructions with some modifications. Tissue sections were deparaffinized using standard methods. Ovarian sections were identified under low power magnification then circled with a paraffin pen to isolate them (Mini PAP Pen No. 6056, Newcomer Supply). 10 µl of Proteinase K (20 µg/mL) was applied to each encircled section and incubated for 15 minutes at RT. Sections were washed twice for 2 minutes with PBS in a coplin jar. Excess liquid was tapped off of the slide and 10 µl of kit Equilibration Buffer was applied to the section and incubated for 10 seconds at RT. After tapping off excess liquid, 10 µl of Working Strength TdT Enzyme was applied to each section and incubated in a humidified chamber at 37°C for 1 hour. After incubation, the slides were placed in a coplin jar containing Working Strength Stop/Wash Buffer. Slides were agitated for 15 seconds then incubated for 10 minutes at RT. Slides were washed 3x 1 minute in PBS in a coplin jar. Excess liquid was tapped off and 10 µL of RT Working Strength Anti-Digoxigenin Conjugate was applied to each section. Slides were incubated in a darkened, humidified chamber at RT for 30 minutes. Slides were washed in coplin jars 4 times alternating PBS or PBS +0.1% Tween-20 at RT. Excess liquid was tapped off and slides were counterstained with a mounting medium containing 0.5 to 1.0 µg/mL Propidium Iodide. Coverslip was placed with Crystal Mount. Slides were imaged on the Nikon A1 confocol, imaging twelve ovarian slices per time point, covering the first third, the middle, and the last third of the ovary to ensure sampling all follicle types. To classify follicles as apoptotic, primary, secondary, and antral follicles were considered positive if they had ≥4 TUNEL-positive granulosa cells [48], and primordial follicles as positive with ≥1 TUNEL-positive granulosa cell. At least 50 follicles were classified per class. Apoptotic index was determined from 4 ovaries per time point utilizing the following criteria: (1) only follicles containing a visible oocyte were scored, (2) follicle types were differentiated by standard morphology and size ranges, (3) follicles that were difficult to classify were not counted.

**Figure 7:**
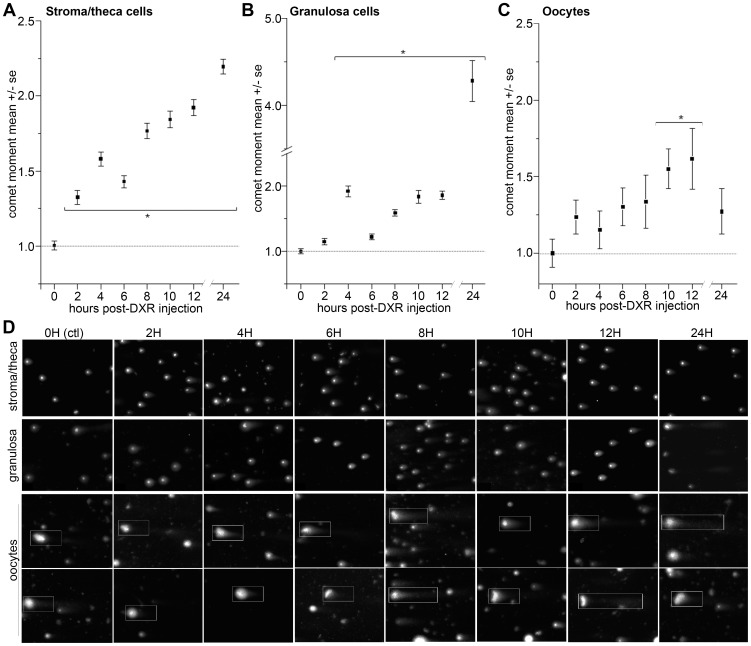
Doxorubicin induced DNA damage in stroma, theca, and granulosa cells by 4 hours post-injection. *A.* Summary graph of DXR-induced DNA damage time line in stroma/theca cells. DXR-induced DNA damage reached detectable levels at 2 hours post-injection as a 30% increase in comet moment (p<0.05, ANOVA, Bonferroni, pooled data from n = 4 exps., 2 mice/point, >100 cells/mouse/point). By 24 hours, DNA damage (comet moment) in stroma/theca cells climbed to over a 2-fold increase compared to control. *B.* Summary graph of DXR-induced DNA damage in granulosa cells plotted vs. time. DXR-induced DNA damage reached detectable levels at 4 hours post-injection as a roughly 2-fold increase in comet moment over control (p<0.05, ANOVA, n = 4 exps., >100 cells/point). Damage was maintained at this level until 24 hours, when cells exhibit a ≥4-fold increase in comet moment reflecting an increase in apoptotic comets. *C.* Summary graph of oocyte comet moments from treated mice showed a gradual increase in DNA damage over time, reaching a 50% increase 10–12 hours post-DXR injection (p<0.05, ANOVA, Bonferroni, n = 4 exps., >30 cells/point). Representative images of comets are shown in lower panel; oocytes are boxed in white to distinguish from granulosa cells.

### Statistics

All experiments were performed in a minimum of triplicate. Graphs and ANOVA analyses were generated using OriginLab. All ANOVAs (one-way and two-way) were conducted including a Bonferroni means comparisons. Data were presented (as mean ±SD), and differences were considered significant at *p*<0.05.

**Figure 8:**
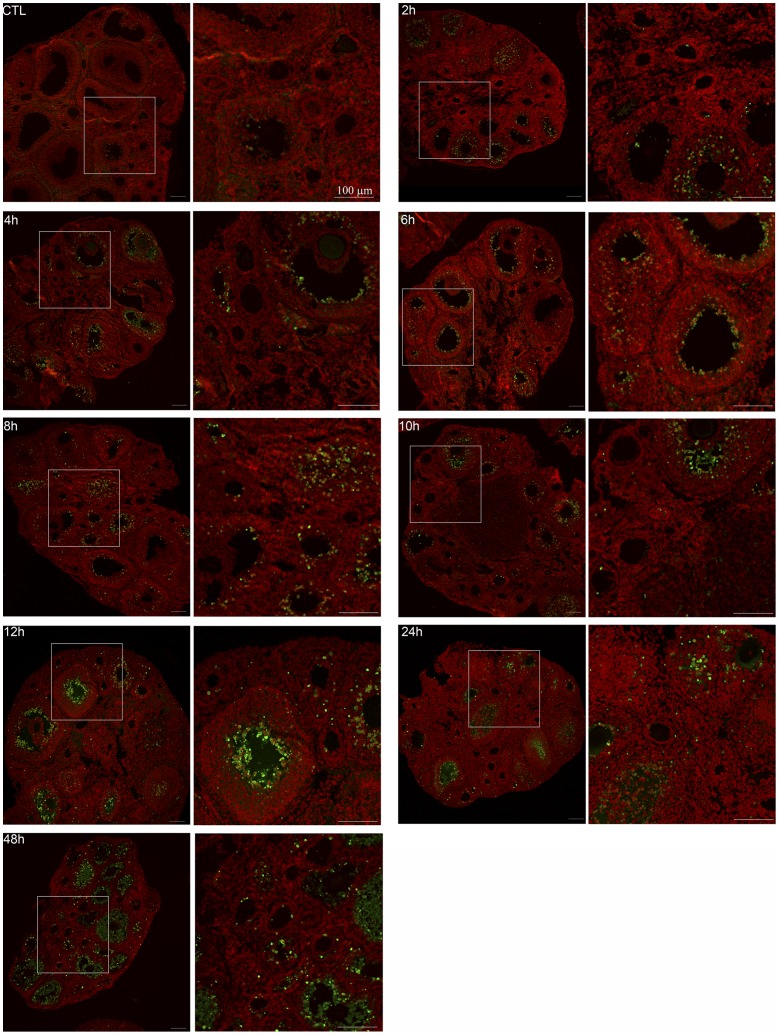
Time-dependent apoptosis following DXR insult. Confocal images show apoptosis-positive cells within the ovary over time following DXR injection. TUNEL-positive cells are shown in green, nuclei in red. Scale bar is 100 µm. Original image is on the left for each time point as labeled, with a digitally-zoomed image on the right. Box in the original image corresponds to the zoomed area.

## Results

### Doxorubicin Exhibits Time-dependent Penetration and Redistribution in the Ovary

To determine the timeline for *acute* doxorubicin (DXR) accumulation and distribution in the ovary, we assessed its appearance and localization in mouse ovaries over a 48-hour period utilizing DXR’s autofluorescence. Representative confocal images of ovarian slices from CD1 mice treated with DXR for 2–48 hours are shown in Fig. 1, with additional examples in Fig. S1. DXR fluorescence (red) was visible 2 hours post-DXR injection, with a notable increase in signal at 4 hours; signal persisted through the 48-hour assay period.

**Figure 9:**
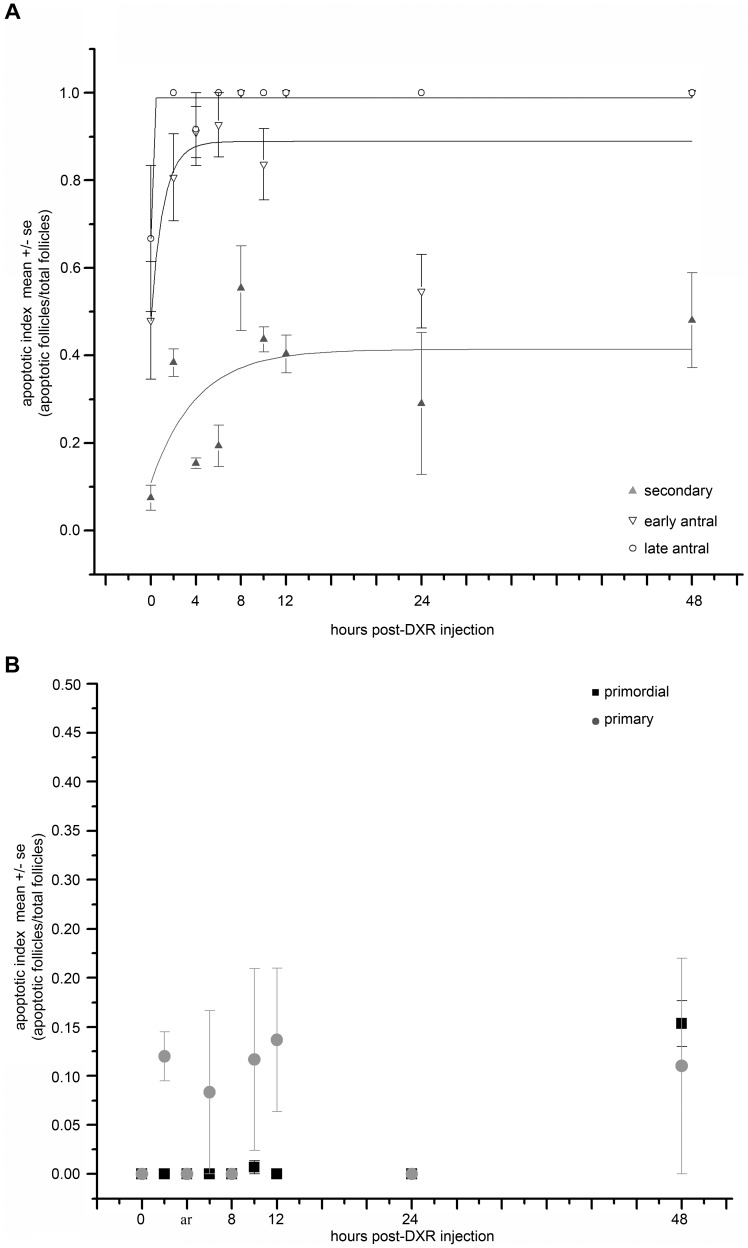
Time-dependent apoptosis following DXR insult is follicle type-dependent. *A.* Graph plots mean apoptotic index for secondary, early antral, and late antral follicles over a 48-hour time period post-DXR injection. *B*. Graph plots mean apoptotic index for primordial and primary follicles over a 48-hour time period post-DXR injection.

### Timeline and Magnitude of DXR-induced DNA Strand Breaks are Ovarian cell-type Dependent

Quantifying total DXR fluorescence in each ovarian section revealed that by 2 hours post-injection, the earliest time point tested, the drug was detectable at ∼4-fold above background (Fig. 2B, p≤0.05, one-way ANOVA). DXR fluorescence measured in the ovarian tissue slices increased to more than 10-fold above background by 4 hours post-injection (Fig. 2A, p≤0.05, one-way ANOVA). From the 4 hour time point through the end of the 48-hour assay period, DXR fluorescence showed no further increase (Fig. 2A).

**Figure 10:**
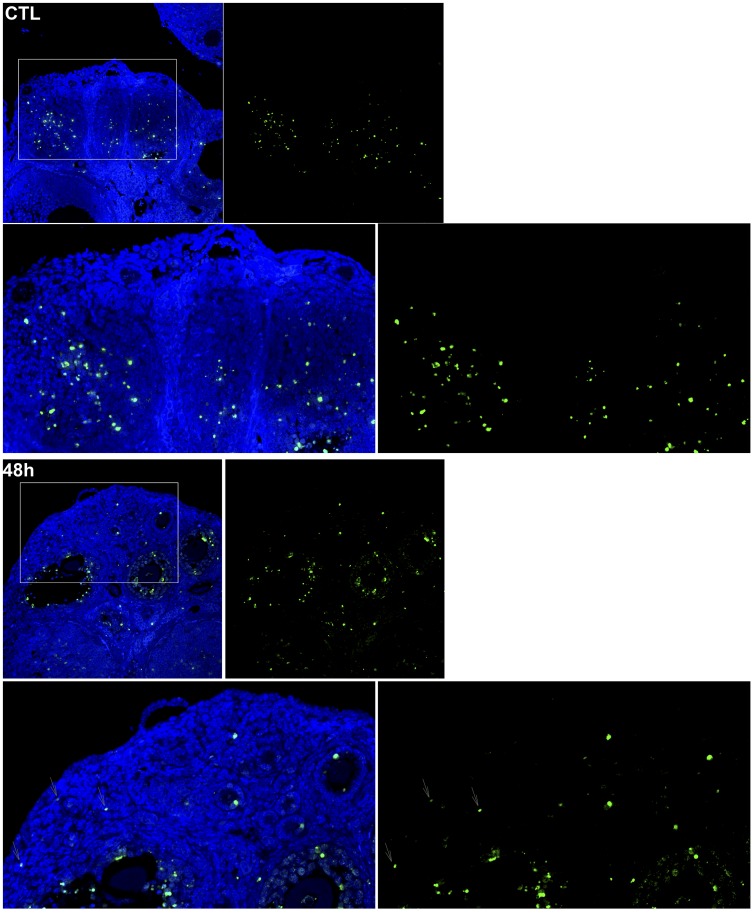
γH2AFX phosphorylation confirms granulosa cells from all follicle classes exhibit dsDNA damage post-DXR insult. Images of confocal sections show cells positive for phosphorylated (activated) γH2AFX (green). Blue signal is DAPI counterstain of the nuclei to provide ovary morphology. γH2AFX phosphorylation is not seen in primordial follicles in the control samples; zoomed areas correspond to box. Arrows indicate phospho-γH2AFX-positive primordial follicles in the images of ovaries harvested 48 hours post-DXR injection. Images are enhanced +15 brightness and +42 contrast to aid in visual clarity (particularly of the DAPI stain) for print.

The distribution of DXR within the heterogeneous structure of the ovary also appeared to change over time. We observed brighter DXR signal in the stroma tissue at early time points (see 4 hour image, Fig. 1), giving the appearance of rings surrounding the dimmer signal in the follicles. Over time, however, the distribution appeared to equilibrate from stroma to follicles, or even become more prominent in follicles than surrounding stroma (see 24 hour image, Fig. 1). We therefore measured the ratio of DXR in the follicles vs. the stromal tissue, normalized to area, to quantify any change in DXR distribution within the ovarian structure over time. This analysis revealed a steady increase in the DXR follicle:stroma ratio through 12 hours post-DXR injection (Fig. 2B). The ratio approached equilibrium, a 1∶1 ratio, from 24–48 hours post-DXR injection (Fig. 2B). These data suggest DXR may redistribute from the stroma and subsequently penetrate into the follicles. Alternatively, DXR levels may remain constant in the cortex and follicles but DXR may be lost from the central stroma due to drug metabolism or stroma cells demise.

We tested the hypothesis that DXR accumulation differs according to follicle class by quantifying mean DXR accumulation in the follicles over time, sorting data according to follicle class. Mean total DXR fluorescence followed an exponential curve over time for each follicular class (Fig. 3A). The larger follicles (secondary, early antral, late antral) accumulated significantly more total DXR than smaller primordial and primary follicles (two-way ANOVA p≤0.05). Follicle size in turn largely depends upon the number of granulosa cells. To determine whether the apparent differences in mean DXR accumulation based on follicle type were due to actual differences in follicular drug penetration or were simply a function of cell number, reflected in follicle size, we normalized the DXR fluorescence to the follicular area. As shown in Fig. 3B, this area normalization minimized the differences in mean DXR fluorescence between follicular classes (ExpDecay fits are given in Table 1). Differences still remained, however, such that the mean amount of DXR fluorescence in primordial and late antral follicles was indistinguishable, but significantly greater than primary, secondary and early antral follicles (two-way ANOVA p≤0.05).

Imaging DXR fluorescent signal at lower magnification revealed time-dependent changes in the drug’s radial distribution across the ovarian sections. At early time points (2, 4 hours), DXR’s fluorescent signal was concentrated at the central core of the ovary, then shifted to a relatively homogenous distribution across the entire ovary section over time (images in Fig. S2). We quantified this radial distribution by taking line profile measurements (fluorescent intensity distributions along a single line) for each confocal section, normalized to peak fluorescent intensity (0 to 1) and distance, such that 0 =  ovary center and 1 =  ovary perimeter (Fig. 4). Plotting the average radial DXR distribution across the ovary at each time point revealed a linear distribution slope of approximately -1.5 for early time points (2 through 8 hours post-injection) (Table 2). At 10–12 hours post-DXR injection, there was an inflection point such that the linear slope stabilized above −0.1 by 24–48 hours post-DXR injection (Fig. 4, Table 2), reflecting the more homogenous DXR distribution. In addition, the radial curves for 4 and 6 hours had a distinctive non-linear shape. These curves became more linear as the slopes simultaneously became less negative and flattened over time post-DXR injection. This was reflected in the bi-dose response curve fit for time points 2–12 hours, which fitted poorly (R^2^≤0.8) at 24 and 48 hours post-DXR injection (Table 2).

### Doxorubicin’s Spectral Profile in the Ovary Changes with Time

We tested the hypothesis that DXR’s subcellular environment changes over time by analyzing the drug’s spectral profile in the ovary via confocal microscopy. Example composite images in which all emission wavelengths are presented layered together as a single image are shown for each time point in Fig. 5. Images in Fig. 5 reveal a relatively homogenous cytosolic distribution for DXR in the stroma cells at all time points. In contrast, the granulosa cells develop distinct perinuclear puncta, or aggregates, in which DXR exhibits a red-shifted emission spectrum. The observed puncta grew larger and more clustered over time, as demonstrated by the dramatic patterns 48 hours post-DXR injection. By 12 hours post- injection, DXR signal in the nucleus also appeared red-shifted (see Fig. S3 for digitally zoomed images).

To quantify changes in DXR’s spectral profile, which would indicate a change in environment (subcellular distribution), we measured the mean spectral profile for DXR over the 48 hour time course of our experiment. Peak intensities for each time point were normalized to 1 to allow cross-comparison of the shape of the spectral curves (fingerprints), and plotted vs. wavelength. Figure 6A shows average curves from control, 4 h and 48 h post-DXR injection (Figure S4 shows curves from all time points). The A1 confocal creates a ‘cold finger’ over which no spectral data are collected so these artifact data points from 550–570 nm were omitted from the graphs. These data demonstrate DXR’s spectral profile was distinct from the autofluorescence in ovarian tissue (Fig. 6A), along with the signal being significantly above threshold (Fig. 2A). In addition, there was a shift in the shape of DXR’s spectral fingerprint over time. Previous spectrophotometry studies have used a peak-to-peak intensity ratio (intensity at 550/intensity at 580) to measure changes in the shape of DXR’s spectral profile [30], [45]. We utilized the emission intensities at 540 nm and 580 nm, on either side of the cold finger, to generate the peak ratio at each time point and plotted this ratio vs. time (Fig. 6B). The peak ratio for DXR in the ovary slices changed over time such that the ratios at 4 hours and 48 hours were different from all other time points, which are boxed in grey (one-way ANOVA, p≤0.05, ≥40 images/time point, n = 4). In addition, both 6 hours and 8 hours were different from 12 hours (one-way ANOVA, p≤0.05, ≥40 images/time point, n = 4), indicating changes DXR’s subcellular environment at these time points.

Utilizing a Gaussian fit, we were able to determine an apparent peak within the cold finger and utilize this as another metric of the changing environment for DXR over time [30]. At 2 hours post-DXR injection, the peak was at 563 nm (Fig. 6C). The peak exhibited a blue shift to 556 nm at 4 hours, gradually moving to longer wavelengths from 6–24 hours (boxed in grey), with a final red shift to 566 nm at 48 hours post-injection (Fig. 6C). These estimated peaks were consistent with the curve shifts and peak ratio changes in Figs. 6A&B, and again indicate the subcellular environment surrounding DXR changed with time.

To assess direct DXR-induced DNA damage prior to the apoptotic response, we utilized the neutral comet assay (NCA) to measure the relative levels of double-strand DNA breaks in granulosa and stroma/theca cells isolated from mice treated with DXR. This sensitive single-cell electrophoretic assay revealed detectable damage in stroma/theca cells as a ∼30% increase (p≤0.05, one-way ANOVA) in comet moment over control values at 2 hours post-injection (Fig. 7A). Damage in the stroma/theca cells continued to rise in a linear fashion throughout the time course assayed, reaching a maximum ∼2-fold increase in DNA damage by 24 hours post-DXR injection. In contrast, granulosa cells first exhibited measurable DNA damage 4 hours post-DXR injection, which was quantified as a ∼2-fold increase in double-strand DNA breaks (p≤0.05, one-way ANOVA) (Fig. 7B). These data indicate granulosa cells exhibit a delayed, but more sensitive DNA damage response than stroma/theca cells, consistent with the drug accumulation timeline. DNA damage levels in the granulosa cells remained steady at an approximate 2-fold increase through the 12-hour time point, jumping to a >4-fold increase over control at 24 hours post-injection (Fig. 7B). Within the 24 hour sample, there was a population of granulosa cells that exhibited an hourglass comet morphology indicative of apoptosis and accounting for the dramatic increase in comet moment. The comet moment measured in oocytes rose relatively slowly, reaching significant 50% increase in DNA damage over control only at 10–12 hours post-injection (Fig. 7C), a comparatively late sequel to stroma/theca and granulosa DNA damage. This slow rise in oocyte DNA damage suggested oocytes were either late targets of DXR or fail as a subsequent result of follicular deterioration. These data define the sites and time course of acute DXR-induced genotoxic insult, providing a window of opportunity to develop intervention therapies to prevent DXR damage in the ovary.

### Doxorubicin-induced Apoptosis in the Ovary

To determine the timeline for cell demise in follicles following DXR injection, we stained ovarian sections for TUNEL as a marker of late-stage apoptosis. Images revealed an increase in TUNEL-positive granulosa cells (green nuclei) over time (Fig. 8, additional images in Fig. S5), with dramatic apoptotic signal throughout the secondary and antral follicle populations by 8 hours post-injection (Fig. 8, quantified in Fig. 9A). To determine how the apoptotic response varied according to follicle type, we calculated the mean apoptotic index as the fraction of TUNEL-positive follicles out of the total follicle count for each class (4 ovaries/time point). DXR induced a roughly 30% increase in the mean fraction of apoptotic follicles for secondary, early, and late antral follicles (Fig. 9A). Primary follicles plateaued with a mean apoptotic index of 12% (Fig. 9b), while apoptotic events in primordial follicles were not detected until 48 hours post-DXR when they reached a mean apoptotic index of 10% (Fig. 9B), despite significant DXR accumulation by 4 hours post-injection (Fig. 3). These data demonstrate acute DXR insult causes earlier and more extensive the demise of growing follicles compared to primordial follicles. We did not identify the germinal vesicle within enough oocytes to confidently assess apoptotic state of individual oocytes; this limitation was dependent upon which slices were analyzed for TUNEL. Interestingly, the number of apoptotic granulosa cells per follicle appears to rise continuously over time in all growing follicle classes, as indicated by the dramatic TUNEL signal at 48 hours (Fig. 8). The number of apoptotic follicles does not continuously rise (Fig. 9), however, suggesting the early commitment to apoptosis within the granulosa cells predicts the overall failure for a given follicle.

### γH2AX Phosphorylation Reports DNA Damage in Primordial Follicles

Since we did not observe TUNEL-positive granulosa cells in primordial follicles until 48 hours despite relatively high levels of DXR accumulation, we stained ovarian sections for phosphorylated γH2AFX to determine whether primordial follicles exhibit DXR-induced DNA damage. γH2AFX is activated (phosphorylated) in response to double-strand DNA breaks, and is one of the earliest cellular reporter of such damage. Prior to 48 hours post-DXR injection, granulosa cells positive for phospho-γH2AFX were detected throughout primary, secondary, and antral follicles, but none was seen in primordial follicles until 48 h (see Fig. S6). Figure 10 shows representative confocal images from control and 48 hours post-DXR injection. Phospho-γH2AFX signal is seen in antral follicles of control samples which contain relatively rapidly dividing granulosa cells; this is expected as normal DNA replication in dividing cells involves topoisomerase-II mediated strand breaks and transient H2AFX activation. Activated γH2AFX is not seen, however, in smaller follicles within the control samples. Arrows highlight DXR-induced phospho-γH2AFX foci in granulosa cells of primordial follicles 48 hours post-injection. Phospho-γH2AFX foci are also evident in primary and secondary follicles 48 hours post-injection, but not seen in control samples. These data demonstrate that primordial follicles exhibit a DNA damage response to DXR insult at 48 hours, but this response is delayed compared to the other follicular classes.

## Discussion

This study defined the timeline of acute DXR accumulation, DNA damage, and subsequent cell death in the mouse ovary in both a cell type- and follicle type-dependent manner. These temporal data provide a necessary framework for the future design of chemoprotective agents specific for the ovary. Previous studies have elegantly identified protective agents that prevent radiation, doxorubicin, or cisplatin-induced apoptosis in the ovary [49], [50], [51]. Apoptosis, however, is a late sequel to the initial ovarian insult. Understanding acute chemotherapy insult allows potential development of intervention approaches to *prevent* initial ovarian injury and maintain oocyte fidelity. We previously identified dexrazoxane as a putative ovarian protective agent for DXR insult *in vitro* [47]. Determining the capacity of dexrazoxane or other agents to protect the ovary *in vivo* requires generating a model of acute chemotherapy insult to provide metrics for assessing protection. We have identified the first 2 hours post-DXR treatment as critical to prevent induced DNA damage in ovarian stroma cell-enriched populations, which maintain the overall structural integrity of the mouse ovary, and are the first targets of DXR insult. Granulosa cells may have up to a 4-hour protection window, as DXR-induced DNA damage in granulosa cell-enriched population is not measurable until 4 hours post-injection. These quantifiable metrics, along with the detailed timeline for drug accumulation and the apoptotic response, provide a framework in which we can assess the efficacy of putative ovarian chemoprotective agents. Understanding and preventing the insult, rather than subsequent cellular demise (apoptosis), may lead to the development of ovarian protective therapeutics that will successfully translate to the clinic.

DXR time-dependent localization appeared coupled to the time line for induced DNA damage. DXR was detected via microscopy in the mouse ovary by 2 hours post-injection based on its autofluorescence, and appeared most prevalently in the core stroma cells at early (2- and 4-hour) time points (Figs. 1,2,4). Consistent with that observation, DNA damage in the stroma-enriched cells was also detected at the 2 hour time point using the comet assay (Fig. 8). DNA damage did not reach detectable levels in the granulosa-enriched cells until 4 hours post-injection, however (Fig. 8), and while the drug was visible in follicles by 2 hours, there was a time-dependent exponential increase in drug within the follicles from 2 to 4 hours (Fig. 3). These data suggest that DXR must penetrate follicles and reach sufficient nuclear concentrations before DNA damage occurs in granulosa cells. This model indicates a slower timeline for granulosa cell damage compared to stroma cells and is consistent with the intimate interaction between stroma cells and the blood supply.

On a daily basis, the ovary must successfully process and detoxify numerous environmental toxins to ensure viable offspring. Our study suggests one basic function of the core ovarian stromal tissue may be to shield follicles from such insults by actively sequestering toxins like DXR. The largest vessels in the ovary are located centrally in the core of the organ and radiate out, but the capillary network infiltrates the entire organ, central medulla to cortex, to ensure rapid gas and nutrient exchange throughout the ovary. Despite this typical organ capillary network, the central core ovarian stromal tissue concentrates DXR at early time points (2–4 hours post-DXR injection). The observed gradient is different from the homogenous DXR distribution observed in the canine prostate following i.p. injection, suggesting ovarian-specific DXR uptake [52]. This indicates that the stromal cells may sequester DXR, which is one way to shield follicles in the face of toxic insult. Though most chemoprotection work to date has focused on follicles and oocytes, previous studies have indeed shown ovarian stroma cell disarray or reorganization following chemotherapy [53], [54], consistent with severe insult to this tissue. The stroma cells may therefore be a key protection site in strategies to prevent chemotherapy insult in the ovary. We did not see wide-spread apoptosis in stoma cells despite dramatic degeneration of the core stromal tissue, suggesting the tissue is destroyed via other mechanisms like necrosis. These data are consistent with a study demonstrating that while ovarian ovulation returns at one month post-DXR treatment in mice, the ovary size does not recover and remains at only 50% normal weight and volume [23]. The decreased ovarian size persists despite measurements showing counts for each follicle class, except secondary, were not different from control at 1 month post treatment [23]. Taken with our data, the measurements by Ben-Aharon et al suggest a dramatic loss of stroma tissue that is not recovered post-treatment. The stroma cells are critical to ovarian structure and follicular recruitment as they regulate the collagen structure surrounding follicles; alterations in the collagen structure in turn affects primordial follicle recruitment [55], [56], [57], [58], [59], [60]. It is therefore possible that one mechanism contributing to long-term chemotherapy-induced ovarian insufficiency is activated reprogramming of primordial follicle recruitment as a consequence of stroma remodeling.

In contrast to stroma cells, granulosa cells appeared to package DXR into discrete puncta, or foci, which then aggregated over time. While the present study did not identify these subcellular compartments, DXR and other chemotherapy agents are found concentrated in perinuclear puncta identified as acidic organelles [61], [62], [63], [64], [65], [66], [67], [68], [69] including lysosomes [68], [70], [71], endosomes [68], and the Golgi [65], [68], [72], depending on the cell type. It is unclear whether these structures play a role in the apparent sensitivity of granulosa cells to DXR-induced DNA damage or are in fact a defense mechanism. One frequently observed feature of multidrug resistant cancer cells is compartmentalization of DXR and other chemotherapy agents into acidic intracellular compartments [31], [35], [61], [63], [65], [66], [67], [68], [69], [70], [71], [72], [73], [74], [75], [76], [77], [78], [79], [80], [81], [82], [83], [84], [85], [86], [87], [88], [89], [90], [91], [92], [93], [94], [95], [96], [97]; this compartmentalization is directly linked to limiting nuclear accumulation of DXR and therefore decreasing chemotherapy toxicity. Exceptions exist, however, such as the uterine drug-sensitive MES-SA cell line which accumulates DXR in lysosomes where the drug-resistant MES-SA/Dx5 cell line does not [76]. The sensitivity to DXR-induced DNA damage exhibited by granulosa cells may reflect the observed subcellular compartmentalization. Alternatively, the granulosa cells in growing follicles may be highly sensitive because they are more rapidly dividing than stroma cells. Rapid cell division and DNA replication are factors that increase DXR-induced DNA damage. Future studies may test the hypothesis DXR accumulates in lysosomes or other similar compartments in granulosa cells, and further determine whether the drug compartmentalization alters granulosa cell sensitivity to chemotherapy.

Oocytes exhibited a much slower rise in DNA damage than either the stroma or granulosa cells, reaching a 50% increase in DNA damage only at 10–12 hours post-DXR injection. This suggests either the surrounding tissue functions to actively protect the oocyte or reflects the relative transcriptional quiescence of the oocytes compared to granulosa cells. A study by Bar-Joseph et al detected DXR in oocytes from secondary follicles at 3 hours post-DXR injection [26], which would suggest oocyte quiescence or resistance may account for the delayed damage response. By 10–12 hours post-injection, there is significant DXR-induced apoptosis in the granulosa cells of growing follicles (Fig. 10, [23]) past the threshold previously defined to signify an apoptotic follicle [25], [26], [48], [98]. Taken together, these data suggest the oocytes may not exhibit a DNA damage response to DXR until the follicle commences apoptosis and hence oocyte attrition is secondary to follicular failure.

The time-dependent spectral changes we observed in this study suggest DXR fluorescence is environmentally sensitive in the ovary and may reflect changes in the dielectric constant and/or pH surrounding DXR [30], [43]. Ongoing studies will be required to define the specific subcellular distribution of DXR in the ovary and how that changes with time. DXR’s fluorescence spectrum has a parabolic dependence upon the dielectric constant of its solvent such the ratio of the 555 nm/590 nm peak intensities from DXR in solution is high in water (∼0.8) [30]. This peak ratio drops in solvents with decreasing dielectric, manifest as a “blue shift,” or a curve shift to shorter wavelengths, with decreasing dielectric constant. In heptane, however, which has the lowest dielectric tested, the peak ratio increases to over 1, manifest as a “red shift” in the peak emissions [30]. DXR’s peak ratio is also dependent upon pH, such that the ratio increases (blue shift) with increasing pH. Our data show that from 2 to 4 hours post-DXR, the spectral profile exhibits a dramatic blue shift that begins to correct back toward longer wavelengths from 6 hours post-injection through a dramatic red shift at 48 hours. These data suggest that between 2 and 4 hours post-injection, DXR may experience a change in environment and become compartmentalized in cellular structures with a decreased dielectric constant or increased pH. The continual ‘red shift’, or increase in peak emissions ratio from 4 hours post-injection through 48 hours could be due in part to the observed DXR accumulation in puncta within the granulosa cells, which may correspond to acidic organelles like lysosomes, with a pH of roughly 5. Future studies may define time-dependent subcellular localization of DXR to organelles with an acidic pH, including endosomes, lysosomes, and the Golgi. The dramatic ‘red shift’ at 48 hours may also be due to the ‘red’ nuclear DXR signal which becomes consistent at this time point. Intercalation into DNA can quench DXR fluorescence up to 80%, and indeed while we see significant DNA damage in both stroma and granulosa cells at early time points (2 hours post-injection and on), we do not consistently observe ‘red’ nuclei until at least 12 hours post-DXR injection. These data suggest a model in which DXR rapidly intercalates DNA, quenching its fluorescence, until all DNA binding sites are occupied. DXR may then accumulate in the nucleus as free drug, accounting for the observed red nuclear fluorescence at later time points.

It was surprising to find that both the late antral follicles and primordial follicles accumulated significantly more DXR per area than the remaining follicle classes. It is likely there are two different mechanisms accounting for what appears to be a similar phenomenon in the two disparate follicle classes. Primordial follicles consist of a single layer of flattened granulosa cells and lack a theca ring. It is possible this single layer (small size) makes it relatively easy for DXR to penetrate throughout the follicle. Late antral follicles are, in contrast, very large. They are also comparatively metabolically dynamic, which may account for the relatively high levels of DXR uptake. Our data suggest DXR uptake was partly dependent upon follicle size, and therefore follicular cell numbers. Additional differences in drug accumulation between follicle classes remained, however, which may reflect unique metabolic activities of the different follicle classes.

Our data demonstrate that within the acute phase of DXR insult, granulosa cells of the secondary and antral follicles are the predominant sites of induced apoptosis. These data are consistent with the study by Ben-Aharon et al showing TUNEL positive granulosa cells predominantly in the antral follicles at 12 and 24 hours post-DXR injection in the mouse ovary [23]. The authors also stated there was weak TUNEL signal in primary follicles [23], which may be consistent with our observation that DXR induced apoptosis in 10% of primary follicles over the time course assayed. Our data include earlier time points, however, which indicate the DXR-induced follicular apoptosis approached plateau in the acute state as early as 8 hours post-injection. This reflects the number of follicles with sufficient apoptotic granulosa cells to predict follicular demise; the number of TUNEL-positive granulosa cells in each follicle, however, continues to rise dramatically through the 48 hour time point, consistent with follicular failure. Our data highlight the necessity of understanding the initial insult and targeting intervention therapies as early as possible to prevent follicular demise. Primordial follicles did not exhibit TUNEL signal nor H2AFX phosphorylation until 48 hours post-DXR injection, despite the relatively high levels of DXR accumulation. This may reflect the relatively quiescent state of the primordial follicles as DXR-induced DNA damage requires DNA replication. Future studies will determine whether the PFs will exhibit delayed DXR-induced apoptosis as they are recruited to grow and the granulosa cells must replicate DNA. Our data propose a model in which primordial follicles sustain DXR injury by 48 hours post-injection, but may not demise until they are triggered to grow.

Delayed apoptosis in the primordial follicles also suggests a “burn out” model may be one mechanism by which DXR depletes the primordial reserve. The burn out model has been proposed for other chemotherapy agents that appear to eliminate the growing follicle population, resulting in increased primordial follicle recruitment to replace the larger follicles [4], [99], [100]. This indirect mechanism depletes the ovarian reserve as primordial follicles are recruited to replace the ‘burnt out’ growing follicles [101]. Taken together, the data in this study demonstrate DXR insult in the ovary is a complex process involving differential effects that are cell- and follicle-type dependent. The data highlight the need to understand acute insult mechanisms and suggest ovarian depletion may be the summation of multiple events including granulosa cell packaging of DXR, stroma demise and remodeling, and direct toxicity in primordial follicles as they are recruited to grow. Future studies may also determine how primordial follicle recruitment after burn out of growing follicles by DXR contributes to depleting the ovarian reserve.

## Supporting Information

Figure S1 **DXR fluorescence in the ovary.** Representative confocal images of DXR signal in ovarian sections reveal time-dependent penetration of DXR into the ovary. Grey signal corresponds to DXR. Images from ctl and mice treated for 2, 4, 6, 8, 10 12, 24 hours with 20 mg/kg were taken using a 20X objective, exciting at 488 nm and collection emissions from 570–620 nm. All images were adjusted +10 brightness and +10 contrast to enhance visibility in print. Scale bar  = 20 µm.
(TIF)

Figure S2 **Radial distribution of DXR in the ovary.** Representative confocal images of DXR signal in ovarian sections reveal time-dependent redistribution of DXR into the ovary. Grey signal corresponds to DXR. Images from ctl and mice treated for 2, 4, 6, 8, 10 12, 24 hours with 20 mg/kg were taken using a 10X objective, exciting at 488 nm and collection emissions from 570–620 nm. All images were adjusted +10 brightness and +10 contrast to enhance visibility in print.
(TIF)

Figure S3 **DXR spectral images.** Example spectral composite confocal images are shown for 12 hours and 48 hours post-DXR injection. Images were generated by exciting at 488 nm and collecting the emissions images every 10 nm from 520 to 700 nm using a 60X objective. All wavelengths are represented in composite images which are digitally zoomed and adjusted +5 brightness and +25 contrast to aid in visualizing puncta.
(TIF)

Figure S4 **DXR’s spectral profile in the ovary shifts over time.** Graphs plot spectral fluorescence intensity values for control ovary images and images from mice treated with DXR at all time points as indicated on graphs. Red line represents Gaussian fit of the data; a grey vertical line is drawn to mark the wavelength at which the curve peaks. The gap in the connecting lines corresponds to the “cold finger” (550–570 nm), or the wavelengths over which the A1 confocal collects no spectral data. Plot reveals shifts in the apparent peak over time post-DXR injection.
(TIF)

Figure S5 **Time-dependent apoptosis following DXR insults is follicle-dependent.** Confocal images show apoptosis-positive cells within the ovary over time following DXR injection. TUNEL-positive cells are shown in green, nuclei in red. Scale bar is 100 µm. Original image is on the left for each time point as labeled, with a digitally-zoomed image on the right. Box in the original image corresponds to the zoomed area.
(TIF)

Figure S6 **γH2AFX phosphorylation confirms granulosa cells from all follicle classes exhibit dsDNA damage post-DXR insult.** Confocal images show phospho-γH2AFX-positive cells (green) within the ovary over time following DXR injection. Nuclei are counter-stained with propidium iodide in red. Scale bar is 100 µm. Green images (γH2AX) are adjusted +30 contrast and merged (red/green) images are adjusted +25 contrast to increase signal visibility in print and on the screen.
(TIF)
