# Supplementary figures and images for: Acute Doxorubicin Insult in the Mouse Ovary Is Cell- and Follicle-Type Dependent
Source: PLoS One. 2012 Aug 2;7(8):e42293. doi: 10.1371/journal.pone.0042293 (PMC3410926; doi:10.1371/journal.pone.0042293)

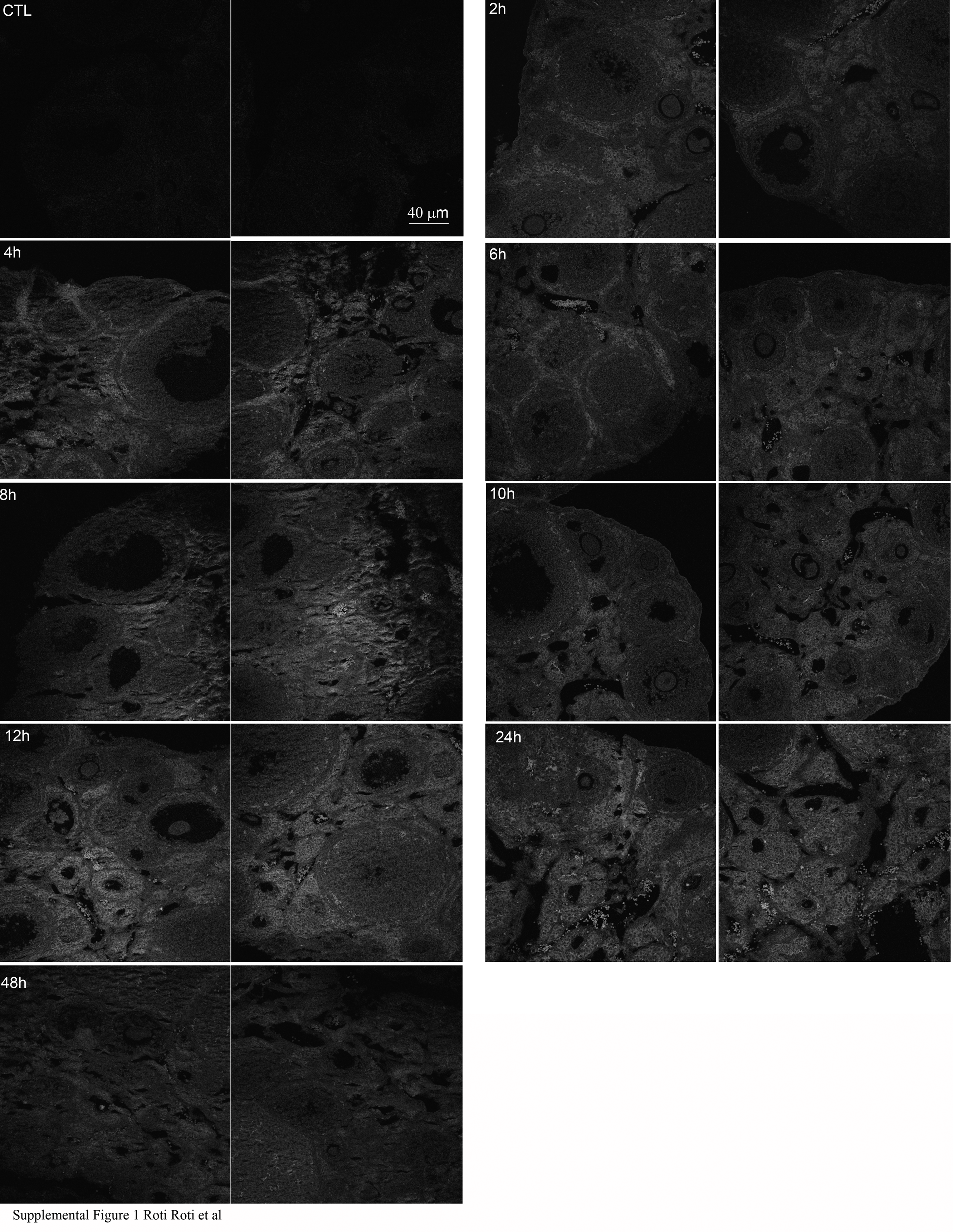

Supplement: Figure S1 — DXR fluorescence in the ovary. Representative confocal images of DXR signal in ovarian sections reveal time-dependent penetration of DXR into the ovary. Grey signal corresponds to DXR. Images from ctl and mice treated for 2, 4, 6, 8, 10 12, 24 hours with 20 mg/kg were taken using a 20X objective, exciting at 488 nm and collection emissions from 570–620 nm. All images were adjusted +10 brightness and +10 contrast to enhance visibility in print. Scale bar = 20 µm. (TIF) [file pone.0042293.s001.tif]

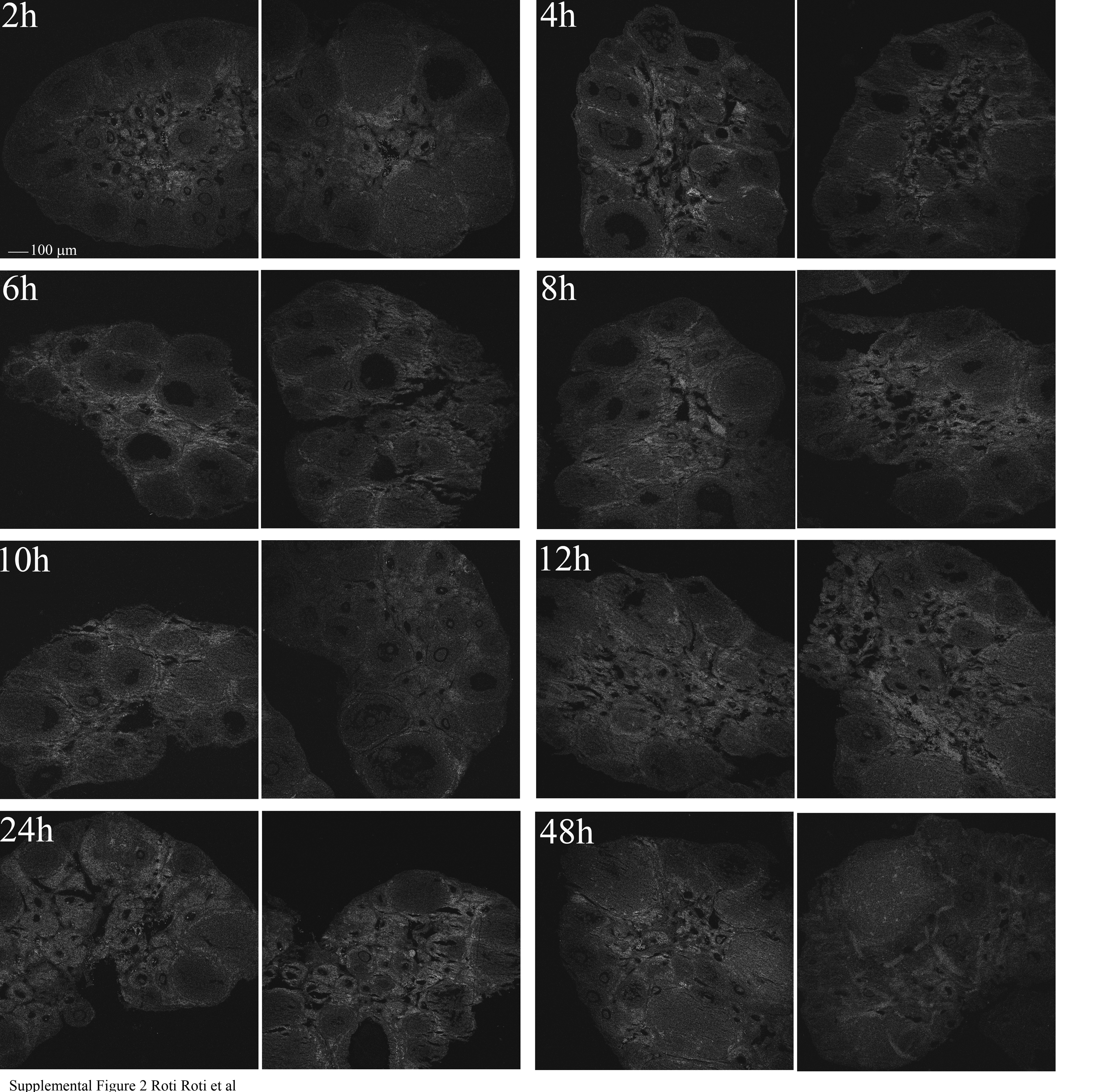

Supplement: Figure S2 — Radial distribution of DXR in the ovary. Representative confocal images of DXR signal in ovarian sections reveal time-dependent redistribution of DXR into the ovary. Grey signal corresponds to DXR. Images from ctl and mice treated for 2, 4, 6, 8, 10 12, 24 hours with 20 mg/kg were taken using a 10X objective, exciting at 488 nm and collection emissions from 570–620 nm. All images were adjusted +10 brightness and +10 contrast to enhance visibility in print. (TIF) [file pone.0042293.s002.tif]

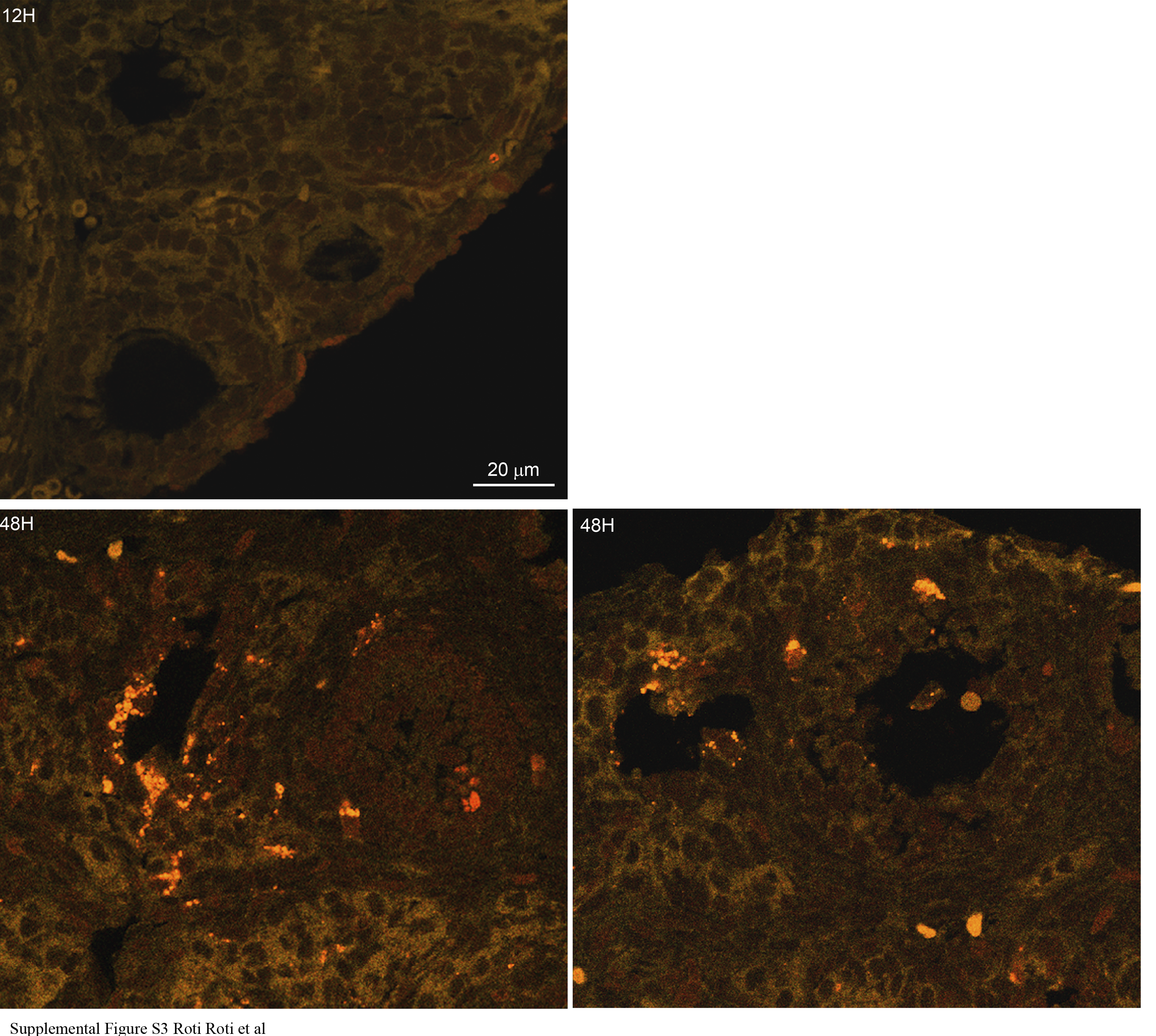

Supplement: Figure S3 — DXR spectral images. Example spectral composite confocal images are shown for 12 hours and 48 hours post-DXR injection. Images were generated by exciting at 488 nm and collecting the emissions images every 10 nm from 520 to 700 nm using a 60X objective. All wavelengths are represented in composite images which are digitally zoomed and adjusted +5 brightness and +25 contrast to aid in visualizing puncta. (TIF) [file pone.0042293.s003.tif]

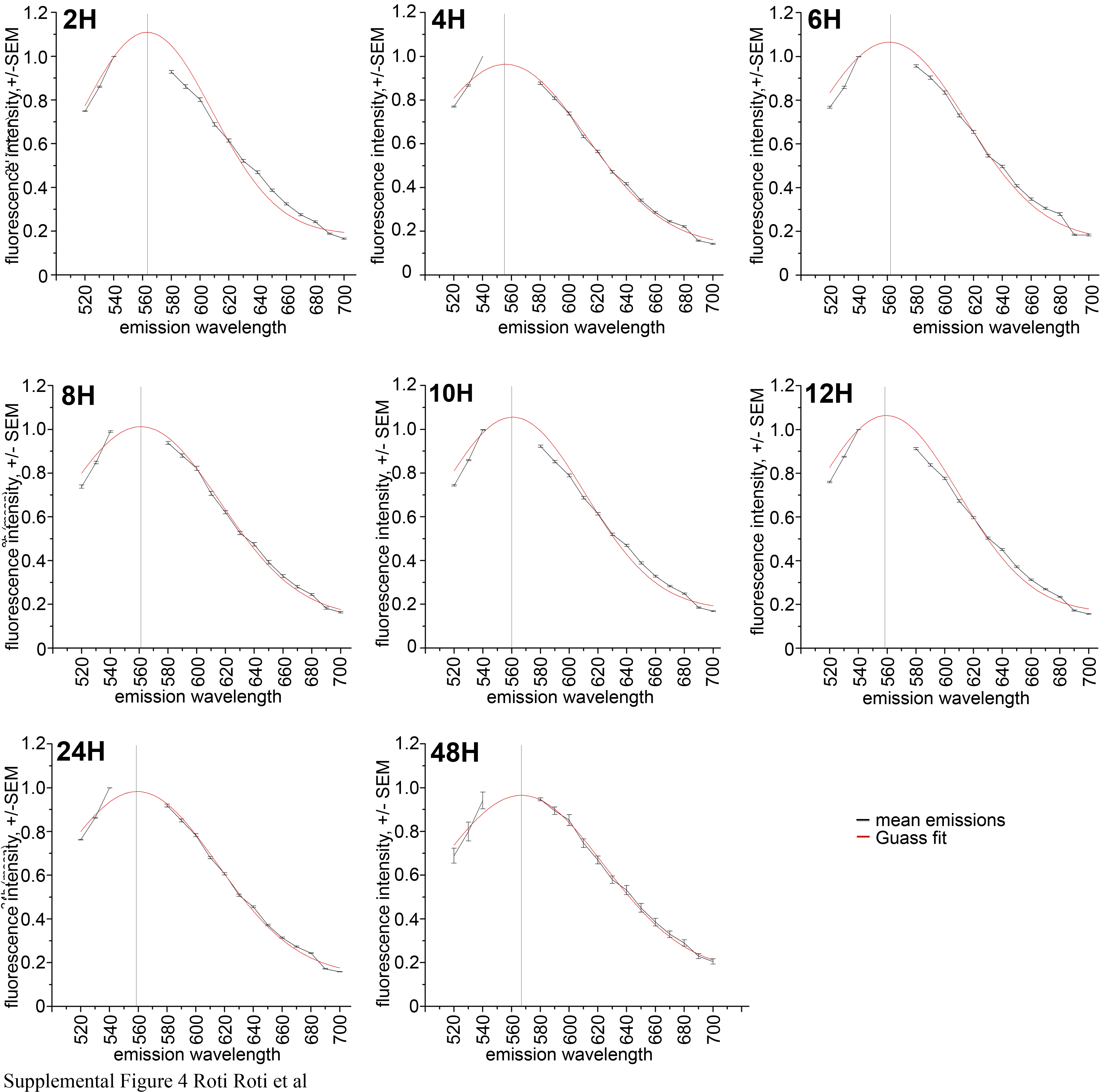

Supplement: Figure S4 — DXR’s spectral profile in the ovary shifts over time. Graphs plot spectral fluorescence intensity values for control ovary images and images from mice treated with DXR at all time points as indicated on graphs. Red line represents Gaussian fit of the data; a grey vertical line is drawn to mark the wavelength at which the curve peaks. The gap in the connecting lines corresponds to the “cold finger” (550–570 nm), or the wavelengths over which the A1 confocal collects no spectral data. Plot reveals shifts in the apparent peak over time post-DXR injection. (TIF) [file pone.0042293.s004.tif]

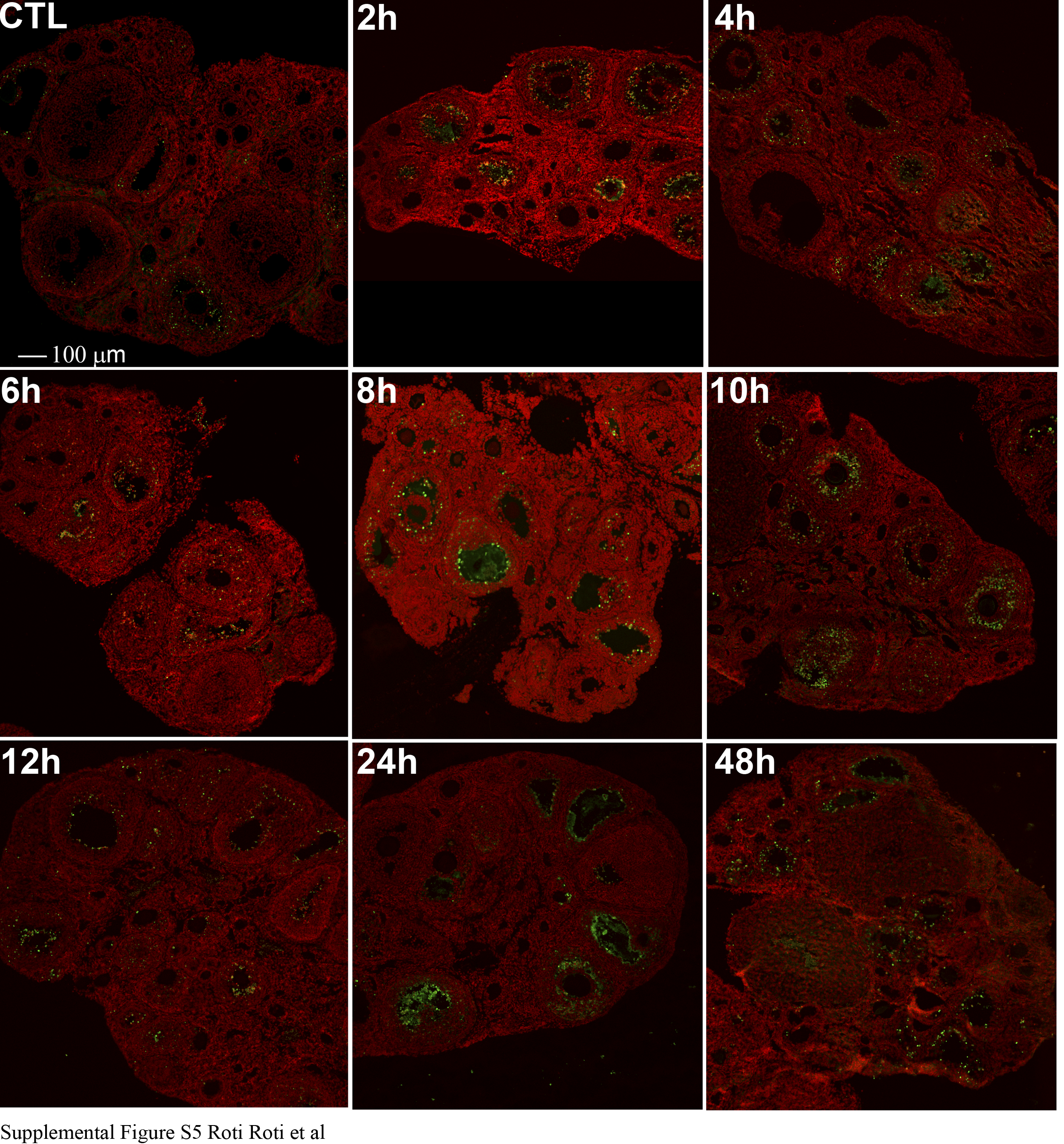

Supplement: Figure S5 — Time-dependent apoptosis following DXR insults is follicle-dependent. Confocal images show apoptosis-positive cells within the ovary over time following DXR injection. TUNEL-positive cells are shown in green, nuclei in red. Scale bar is 100 µm. Original image is on the left for each time point as labeled, with a digitally-zoomed image on the right. Box in the original image corresponds to the zoomed area. (TIF) [file pone.0042293.s005.tif]

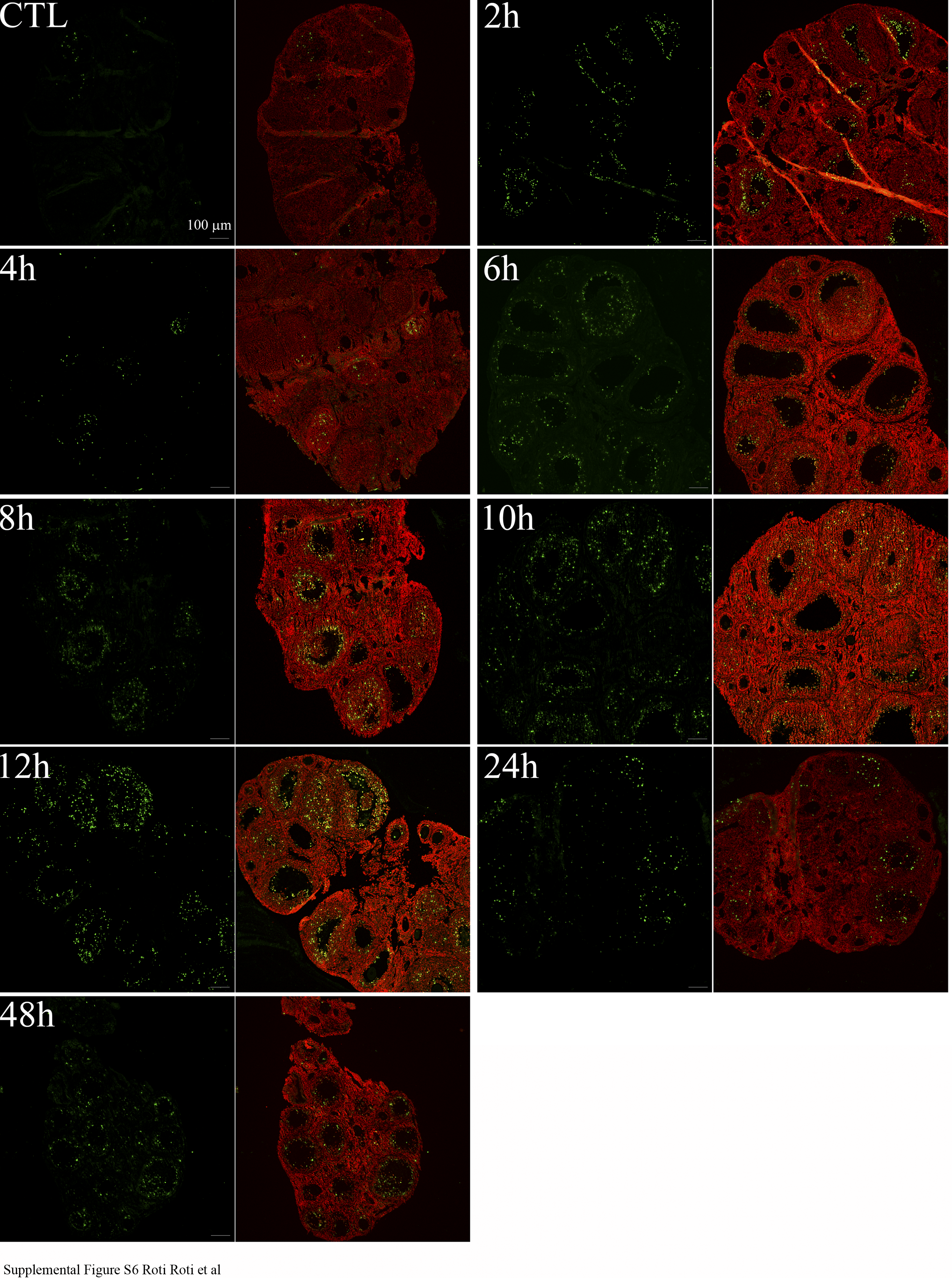

Supplement: Figure S6 — γH2AFX phosphorylation confirms granulosa cells from all follicle classes exhibit dsDNA damage post-DXR insult. Confocal images show phospho-γH2AFX-positive cells (green) within the ovary over time following DXR injection. Nuclei are counter-stained with propidium iodide in red. Scale bar is 100 µm. Green images (γH2AX) are adjusted +30 contrast and merged (red/green) images are adjusted +25 contrast to increase signal visibility in print and on the screen. (TIF) [file pone.0042293.s006.tif]
